# Prostate cancer in men under 50: the impact of race/ethnicity and family history

**DOI:** 10.1080/07853890.2025.2536202

**Published:** 2025-07-21

**Authors:** Lechuang Chen, Yu Zhang, Qing H. Meng

**Affiliations:** Department of Laboratory Medicine, The University of Texas MD Anderson Cancer Center, Houston, TX 77030, USA.

**Keywords:** Family history, Gleason score, prostate cancer, race/ethnicity, younger men

## Abstract

**Background:**

Prostate cancer remains a significant global public health concern. It is the most common cancer and the second leading cause of cancer death among men in the United States. Current guidelines offer varying recommendations on prostate cancer screening, and most are focused on men aged 50–69. In this study, we examined key risk factors for prostate cancer in men under 50, emphasizing race/ethnicity and family history to better understand the distribution of higher-grade disease in this younger population.

**Methods:**

This retrospective analysis utilized data from our hospital’s prostate cancer screening program collected between 2014 and 2024. In our cohort, a total of 312 men under 50 years of age underwent prostate biopsy. We assessed the association of Gleason scores, race/ethnicity, family history, and PSA levels using descriptive statistics analyses.

**Results:**

Among 312 participants, the largest subgroup was White or Caucasian (*n* = 202, 64.7%), followed by Black or African American (*n* = 47, 15.1%), Hispanic or Latino (*n* = 46, 14.7%), and Asian or Pacific Islander (*n* = 17, 5.5%). Black or African American men showed a higher proportion of Gleason 7 and above compared with other racial/ethnic groups. A positive first-degree family history was also more frequent among Black or African American men and was correlated with elevated Gleason scores and elevated PSA levels in multiple racial/ethnic categories.

**Conclusion:**

In our cohort of men under 50, both race/ethnicity and a positive family history are closely associated with higher-grade prostate cancer. These findings suggest that younger men from high-risk backgrounds may benefit from early detection strategies.

## Introduction

Prostate cancer has been identified as the most common male genital cancer among American men and is increasingly recognized as a global public health issue [1]. In the United States, it is particularly regarded as a crisis among African American populations and ranks as one of the leading causes of cancer mortality [2].

Prostate-specific antigen (PSA) is a protein produced by cells in the prostate gland. Elevated PSA levels in a man’s blood may indicate the presence of prostate cancer. Therefore, PSA testing plays a critical role in early detection of prostate cancer [3]. Current practices often use a threshold of 4 ng/mL to trigger further evaluation, PSA levels between 4 and 10 ng/mL, often considered the ‘borderline or grey zone’ have a 25% risk of developing prostate cancer [4], while levels greater than 10 ng/mL indicate an over 50% risk of cancer [5]. Cases with PSA levels exceeding 10 ng/mL also typically warrant further evaluation, including a prostate biopsy.

Although most clinical guidelines focus on men aged 50–69 years, there is a shift in perspective [6–8]. Growing evidence suggests that certain groups under 50 may also be at risk [9,10]. In the Prostate Cancer Early Detection Study Based on a Baseline PSA Value in Young Men trial, screening of ≥45-year-old men revealed that while overall prostate cancer detection rates were low, approximately 70% of screen-detected cancers at this age were clinically significant [11]. The European Randomized Study of Screening for Prostate Cancer has demonstrated that early PSA values strongly predict future risk of aggressive prostate cancer [12]. For younger men with elevated risk factors, a baseline PSA test between ages 40–45 may provide valuable prognostic information to guide subsequent screening frequency [13]. Particularly, Black or African American men experience higher prostate cancer incidence and mortality compared with other racial/ethnic groups [14]. Similarly, men with a strong family history of prostate cancer can have as much as a two- to fivefold increase in risk [15]. Recent study examining men who continued PSA screening past age 70 supports the notion that age alone should not dictate the cessation of screening efforts. This study found that while the overall risk for prostate-cancer–specific mortality was low among men aged 70 and above, screening PSA levels and racial and ethnic backgrounds significantly influenced risk assessments [16]. These findings align with our hypothesis that age criteria for prostate cancer screening need to be more flexible and adapted to individual risk profiles.

With these considerations, we focused our study on how PSA screening correlates with key risk factors—particularly race/ethnicity and family history—among men under the age of 50. By examining Gleason score distributions in this younger population, we try to better understand whether certain risk factors may predict more aggressive disease and how these findings can inform earlier or more personalized prostate cancer screening recommendations.

## Methods

### Study design and setting

This is a single-center, retrospective cohort study of men under 50 years of age who did PSA screening and subsequent prostate biopsy within UT MD Anderson Cancer Center prostate cancer screening program from January 1, 2014, to August 30, 2024. We adhered to the Strengthening the Reporting of Observational Studies in Epidemiology guidelines in designing and reporting this investigation [17].

### Data source and population

All data were extracted from the hospital’s electronic medical records, including laboratory results, clinical notes, and pathology reports. We identified which men participated in our PSA screening program could be included in our study cohort. For each individual, inclusion required:

1. Men under 50 years of age.

2. At least one documented PSA measurement of ≥4 ng/mL, or presence of clinical suspicion based on digital rectal examination, abnormal prostate imaging results, and other clinical concerns identified by clinicians despite lower PSA levels.

3. A subsequent prostate biopsy performed at our institution.

The total number of men enrolled was 339. We then excluded individuals with incomplete medical records, including those who refused to disclose race/ethnicity, lacked a family history, or were diagnosed with prostate cancer but had no Gleason score. After applying these criteria, a total of 312 men with complete laboratory, clinical, and pathological data remained for final analysis.

### Variables and definitions

**PSA screening**: PSA levels were measured using electrochemiluminescence immunoassay on the Roche Cobas system, following the manufacturer’s instructions and the laboratory quality control procedures.

**Prostate biopsy**: All participants had transrectal ultrasound–guided biopsy. Pathology reports provided the histological diagnosis.

**Outcome**: The primary outcome was histologically confirmed prostate cancer. Stratifications included benign and Gleason score category (GS = 6, GS ≥ 7).

**Family history**: ‘Prostate Cancer’ was defined as having at least one first-degree relative (father or brother) with prostate cancer, ‘Cancer’ was defined as family history of any cancer excluding a first‐degree relative with prostate cancer, ‘No Cancer’ was defined as no family members with any type of cancer. All of them are documented by the physician or self-report recorded in the medical chart.

**Race/ethnicity**: Race/ethnicity data were self-reported at participant registration and categorized into: White or Caucasian (Not Hispanic or Latino), Black or African American, Hispanic or Latino, with Asian, American Indian or Alaska Native, and Native Hawaiian or Other Pacific Islander grouped together as Asian or Pacific Islander.

### Data collection and analysis

Information on age at screening, race/ethnicity, family history of prostate cancer, results (including Gleason score) and PSA levels were retrieved. Descriptive statistics were used to characterize the cohort by race/ethnicity, family history, and Gleason score. Categorical variables were summarized using frequencies and percentages. A chi-squared (χ^2^) test and analysis of variance (ANOVA) were performed to assess differences in Gleason scores and family history across various racial/ethnic groups. Subsequently, a Kruskal–Wallis test followed by post-hoc Dunn’s test were performed for each characteristic group (race/ethnicity, family history, and biopsy results) to examine PSA levels. Statistical significance was defined as *p* < 0.05, and high significance as *p* < 0.01.

### Ethical approval and consent to participate

This study was conducted in accordance with the declaration of Helsinki. This study was approved by the Institutional Review Board (IRB) of MD Anderson Cancer Center, the University of Texas (IRB: 2024-0449). All participant data were de-identified prior to analysis, and the study complied with local and national regulations regarding participant privacy and the use of medical data for research. The IRB of The University of Texas MD Anderson Cancer Center determined that the study met the criteria for exemption under Category 4 (secondary research on data or specimens, no consent required), and a waiver of HIPAA authorization was granted for use of PHI.

## Results

**Figure 1.**
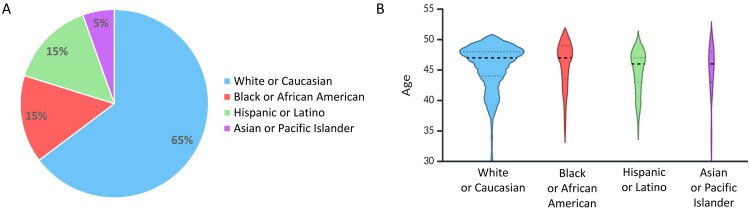
Distribution of different races/ethnicity in the study. (A) The pie chart displays the proportions of participants (*n* = 312) across four racial/ethnic categories: White or Caucasian, Black or African American, Hispanic or Latino, and Asian or Pacific islander. (B) The violin plot displays age distributions for participants in the study. Each plot outlines the density of participants’ ages, with the width representing the frequency at different ages. Dashed lines indicate the quartiles, highlighting the median and interquartile ranges.

Our study included 312 men under 50 years of age, the majority were White or Caucasian (*n* = 202, 64.7%), followed by Black or African American (*n* = 47, 15.1%), Hispanic or Latino (*n* = 46, 14.7%), and Asian or Pacific Islander (*n* = 17, 5.5%) (Figure 1A). The racial distribution of our study cohort also perfectly mirrors the general population composition in the US, which enhances the external validity of our findings [18]. The age ranges for these groups were as follows: White or Caucasian (30–49 years, median = 47), Black or African American (36–49 years, median = 47), Hispanic or Latino (36–49 years, median = 46), and Asian or Pacific Islander (34–49 years, median = 46). The age distribution reflects the composition of individuals who attended our screening program and subsequently underwent prostate biopsy (Figure 1B).

**Figure 2.**
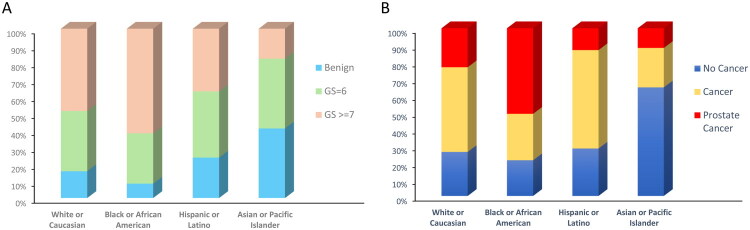
Distribution of prostate cancer characteristics across racial/ethnic groups. (A) Stacked bars represent the proportion of participants in each Gleason score category (Benign, GS = 6, GS ≥ 7) for White or Caucasian, Black or African American, Hispanic or Latino, And Asian or pacific islander men. (A) The stacked bars represent three categories of family history in each racial/ethnic group: ‘No Cancer’ (blue), indicating no family members with any type of cancer; ‘Cancer’ (yellow), indicating family history of any cancer excluding a first‐degree relative with prostate cancer; and ‘Prostate Cancer’ (red), indicating one or more first-degree relative (e.g. father or brother) diagnosed with prostate cancer.

In the study cohort of 312 men, we analyzed their Gleason score distributions and family history status across four self-identified racial/ethnic groups (Table 1). The proportion of each Gleason score category is illustrated by race/ethnicity using stacked bars (Figure 2A). Black or African American men appear to have a higher proportion of high-risk grade tumors (GS ≥ 7, 61.7%) than other groups, compared to their benign (8.5%) and GS = 6 (29.8%) findings. Hispanic or Latino participants have a relatively larger share of GS = 6 (39.1%) and lower incidences of high-risk grades GS ≥ 7 (37.0%). White or Caucasian men show a broader spread across benign findings (15.8%), GS = 6 (35.6%), and high-risk grades GS ≥ 7 (48.5%), while Asian or Pacific Islander men predominantly around benign findings (41.2%) or lower grade GS = 6 (41.2%), with fewer high-risk GS ≥ 7 (17.6%). Distributions across racial/ethnic groups revealed statistically significant differences with a *p* value of 0.013.

**Table 1.** Distribution of Gleason scores and family history by race/ethnicity.

Race/ethnicity	No. of participants	Gleason score			Family history		
		Benign	GS = 6	GS ≥ 7	No Cancer	Cancer	Prostate Cancer
White or Caucasian Not Hispanic or Latino	202	32 (15.8%)	72 (35.6%)	98 (48.5%)	53 (26.2%)	102 (50.5%)	47 (23.3%)
Black or African American	47	4 (8.5%)	14 (29.8%)	29* (61.7%)	10 (21.3%)	13 (27.7%)	24* (51.1%)
Hispanic or Latino	46	11 (23.9%)	18 (39.1%)	17 (37.0%)	13 (28.3%)	27 (58.7%)	6 (13.0%)
Asian or Pacific Islander	17	7 (41.2%)	7 (41.2%)	3 (17.6%)	11 (64.7%)	4 (23.5%)	2 (11.8%)

No Cancer: no relative with any type of cancer; Cancer: family history of any cancer excluding a first‐degree relative with prostate cancer; Prostate Cancer: first-degree relative with prostate cancer. Chi-squared and ANOVA tests were used to evaluate differences in Gleason scores and family history across four racial/ethnic groups. Differences were considered statistically significant at *p < 0.05.

The relationship between the family history of prostate cancer and tumor grade is depicted for each racial/ethnic group (Figure 2B). The stacked bars represent three levels of family history [1]: no known relatives with any type of cancer (‘No Cancer’) [2], family history of any cancer excluding a first‐degree relative with prostate cancer (‘Cancer’), and [3] one or more first-degree relative diagnosed with prostate cancer (‘Prostate Cancer’). The proportions are as follows: for White or Caucasian men, 26.2% reported no cancer, 50.5% reported non-prostate cancer in the family, and 23.3% had a prostate cancer history in first-degree relatives; for Black or African American men, these figures are 21.3% for no cancer, 27.7% for other cancers, and 51.1% for prostate cancer in first-degree relatives; for Hispanic or Latino men, 28.3% had no cancer history, 58.7% had other cancers, and 13.0% had prostate cancer in first-degree relatives; and for Asian or Pacific Islander men, the numbers are 64.7% with no cancer, 23.5% with other cancers, and 11.8% with prostate cancer in first-degree relatives. When viewed alongside the Gleason score patterns from Table 1, a clear trend emerges for certain groups. Particularly, Black or African American men not only show a higher proportion of first-degree family history (red segment) but also a notable presence of higher Gleason scores (as shown in previous figures). These differences (*p* = 0.016) suggest the need for target screening and closer follow-up among such high-risk populations.

In our study, we also examined PSA levels across different racial/ethnic groups, family history, and biopsy Gleason scores, focusing on PSA values categorized by risk level (Normal: PSA < 4, Intermediate: 4 ≤ PSA ≤ 10, and High-risk: PSA > 10) within our cohort (Table 2).

Among racial groups, Black or African American men exhibited the highest overall PSA mean levels (86.9 ng/mL), significantly higher than those observed in White or Caucasian (35.8 ng/mL), Hispanic or Latino (8.0 ng/mL), and Asian or Pacific Islander groups (6.0 ng/mL). This difference was particularly pronounced in the high-risk category, where Black men presented the highest mean PSA level (232.1 ng/mL), compared with White (176.0 ng/mL), Hispanic (18.0 ng/mL), and Asian men (13.4 ng/mL). Consistent with these findings, Black men also had the highest proportion classified in the high-risk PSA category (36.2%), compared with White (18.3%), Hispanic (21.7%), and Asian men (11.8%).

When stratified with family history, men with no reported any cancer history had lower PSA levels (10.0 ng/mL), while those with family history of cancer showed higher PSA levels (38.6 ng/mL). The group with a family history of prostate cancer displayed the highest mean PSA (72.9 ng/mL) and notably elevated levels within the high-risk category (249.4 ng/mL). Further analysis by biopsy characteristics, participants with benign biopsies presented lower mean PSA levels (5.1 ng/mL), while PSA levels increased with biopsy severity. Participants with Gleason scores of 6 had higher PSA means (18.7 ng/mL), and those with GS ≥ 7 showed the highest PSA means (64.9 ng/mL). The observed differences of PSA levels between groups within race/ethnicity, family history, and biopsy Gleason scores were statistically significant.

**Table 2.** PSA levels in participants with different risk categories.

Characteristic	No.	Mean (95% CI)						
		General Total PSA	No.	Normal (PSA < 4)	No.	Intermedia (4 ≤ PSA ≤ 10)	No.	High-risk (PSA > 10)
Race/ethnicity								
White or Caucasian	202	35.8 (14.9–56.7)	57	1.4 (1.0–1.7)	108	5.9 (5.6–6.2)	37	176.0 (69.6–282.4)
Black or African American	47	86.9* (3.7–170.2)	13	1.6 (0.9–2.4)	17	7.0 (6.3–7.7)	17	232.1** (4.4–459.8)
Hispanic or Latino	46	8.0 (5.8–10.3)	10	2.1 (1.3–2.9)	26	6.5 (5.8–7.2)	10	18.0 (10.1–25.8)
Asian or Pacific Islander	17	6.0 (3.9–8.1)	6	1.7 (0.1–3.3)	9	7.1 (6.3–8.0)	2	13.4 (10.9–15.9)
Family history								
No Cancer	87	10.0 (5.8–14.3)	22	1.4 (0.9–1.9)	49	6.2 (5.7–6.7)	16	33.6 (12.9–54.3)
Cancer	157	38.6 (14.3–62.9)	46	1.6 (1.2–2.0)	80	6.1 (5.8–6.5)	31	177.2* (61.9–292.5)
Prostate Cancer	68	72.9* (9.8–135.9)	18	1.4 (0.7–2.1)	31	6.2 (5.7–6.7)	19	249.4** (30.2–468.6)
Biopsy								
Benign	54	5.1 (4.1–6.2)	20	1.8 (1.2–2.5)	29	6.0 (5.3–6.8)	5	13.1 (7.4–18.8)
GS = 6	111	18.7 (2.1–35.2)	28	1.8 (1.4–2.3)	66	6.2 (5.8–6.5)	17	94.9 (15.1–204.9)
GS ≥ 7	147	64.9* (28.2–101.5)	38	1.1 (0.7–1.5)	65	6.2 (5.9–6.6)	44	206.6** (91.9–321.3)

GS, Gleason score; CI, confidence interval; No., No. of Participants. Kruskal–Wallis test was used to evaluate differences across groups followed by post hoc Dunn’s test to compare each pair of groups. Differences were considered statistically significant at *p < 0.05 or **p < 0.01.

Overall, our findings highlight significant variability in the Gleason score distribution and family history across different racial/ethnic groups within our cohort. These characteristics strongly correlate with elevated PSA levels. Black or African American men, in particular, displayed higher proportions of advanced Gleason scores and a more frequent first-degree family history of prostate cancer compared to other racial/ethnic groups.

## Discussion

In this retrospective study of 312 men under 50 years of age who had PSA screening and subsequent biopsy observed, we observed notable differences in Gleason score distribution across racial/ethnic groups and varying levels of family history. Black or African American men were more likely to present with Gleason 7 or higher disease which aligns with previous reports suggesting a disproportionate burden of aggressive prostate cancer in this population [19]. Additionally, the presence of a first-degree relative with prostate cancer appeared to be more common in Black men compared to other groups. Together, these findings emphasize the potential benefits of tailored screening strategies for individuals at higher risk. Our results also showed that White or Caucasian participants constituted the largest group, with a broad range of Gleason scores. Hispanic or Latino men predominantly had lower to moderate Gleason scores (6 or 7), whereas Asian or Pacific Islander men tended to have benign findings or low-grade disease, though a small proportion presented with Gleason 8 or 9. These patterns could be influenced by genetic factors, sociocultural differences in health behaviors, or variations in screening practices [14].

The findings from Table 2 further highlight the clinical correlation of racial/ethnic backgrounds, family history, biopsy characteristics, and elevated PSA levels in younger men. Consistent with previous results presented in our study, Black or African American men under 50 not only had higher Gleason scores but also showed significantly elevated PSA levels, confirming the high risk in this population. In contrast, the Asian or Pacific Islander group presented with lower PSA values and fewer aggressive cases. Additionally, the observed direct association between higher PSA levels and increased Gleason scores emphasizes the importance of PSA screening and testing, potentially allowing for earlier detection and intervention in younger men with high-risk factors.

Although our primary focus was on race/ethnicity and family history, we also reviewed available genetic testing data within our cohort to identify inherited risk factors. Of the 312 participants, 68 underwent BRCA1/2 and high-risk genetic panel testing. Among them, six White men were found to carry BRCA1 or BRCA2 mutations. All six individuals had high-grade disease (Gleason score 7–9) and a documented family history of cancer, including prostate cancer in some cases. However, not all patients in our study had undergone genetic testing. In several cases, testing was discussed but declined due to lack of insurance coverage or because patients did not meet the current NCCN testing criteria. Given the small number of BRCA-positive cases and limited testing coverage across the cohort, these results were not included in our main statistical analysis but offer qualitative support for the role of hereditary cancer syndromes in young-onset prostate cancer.

Several strengths enhance the relevance of our findings. First, all participants in the study had definitive biopsy data, thereby reducing uncertainty regarding the true presence or absence of prostate cancer. Second, the detailed collection of race/ethnicity and family history information provided a robust framework for examining risk factors. However, our study also has limitations. First, prostate cancer screening was performed in cancer hospitals in participants primarily with a high suspicion of cancer of referred in rather than individuals undergoing routine screening. Additionally, this study is single-centered and retrospective, which may not fully represent diverse populations. Future studies would benefit from a multicenter approach. Another limitation is that our cohort was relatively small for certain subgroups, particularly Asian or Pacific Islander men. Future study will require larger and more diverse sample sizes. The next limitation is race/ethnicity and family history were based on self-reported data, which may introduce recall bias. This should be considered when interpreting associations between these factors and prostate cancer risk. Furthermore, we focused on a specific age group that the cohort individuals are under 50. Consequently, they might have potentially higher health awareness or distinct genetic predispositions. The findings of this study should be cautiously applied to the broader population of all men across different age groups.

To maximize the diagnostic utility of cancer screening, a risk-based or ‘targeted’ screening approach should be considered. This strategy might minimize unnecessary biopsies and reduce the risk of overdiagnosis. In addition to race/ethnicity and family history, other factors such as inherited genetic mutations may also change a patient’s overall risk [20]. By combining these factors in a predictive model—potentially enhanced by artificial intelligence (AI)—clinicians could more accurately determine the most appropriate age to begin screening, the frequency of follow-up, and the optimal method for performing targeted biopsies. This more personalized approach can help reduce false-positive results and biopsy-related harms by targeting men who are most likely to benefit from early treatment.

In conclusion, while prior studies have acknowledged that race/ethnicity and family history are risk factors for prostate cancer, most research has focused on older populations (>50 years). Our study is one of the few to investigate how these risk factors interact specifically in men under 50. Our findings demonstrate that Black or African American men and individuals with a strong family history are at increased risk for more aggressive forms of prostate cancer. These results support current clinical guidelines that recommend earlier and more frequent evaluations for high-risk individuals. Integrating relevant risk factors into model-based or AI-assisted screening algorithms may enhance the accuracy and early detection of clinically significant prostate cancers and ultimately improve treatment outcomes for younger men at elevated risk.

## Data Availability

The data supporting the findings of this study is available from the corresponding author upon reasonable request.
